# Evaluation of significant genome-wide association studies risk — SNPs in young breast cancer patients

**DOI:** 10.1371/journal.pone.0216997

**Published:** 2019-05-24

**Authors:** Michelle Rath, Qiyuan Li, Huili Li, Sara Lindström, Alexander Miron, Penelope Miron, Anne E. Dowton, Meghan E. Meyer, Bryce G. Larson, Mark Pomerantz, Ji-Heui Seo, Laura C. Collins, Hilde Vardeh, Elena Brachtel, Steven E. Come, Virginia Borges, Lidia Schapira, Rulla M. Tamimi, Ann H. Partridge, Matthew Freedman, Kathryn J. Ruddy

**Affiliations:** 1 Department of Medical Oncology, Dana-Farber Cancer Institute, Boston, United States of America; 2 National Engineering Research Center for Biochip, Shanghai Biochip Limited Corporation, Shanghai, China; 3 Department of Epidemiology, University of Washington, Seattle, United States of America; 4 Public Health Sciences Division, Fred Hutchinson Cancer Research Center, Seattle, United States of America; 5 Department of Genetics and Genome Sciences, Case Western Reserve University, Cleveland, United States of America; 6 Department of Pathology, Beth Israel Deaconess Medical Center, Harvard Medical School, Boston, United States of America; 7 Department of Pathology, Massachusetts General Hospital and Harvard Medical School, Boston, United States of America; 8 Beth Israel Deaconess Medical Center, Boston, United States of America; 9 University of Colorado Denver, Aurora, United States of America; 10 Stanford University Medical Center, Palo Alto, United States of America; 11 Department of Epidemiology, Harvard T.H. Chan School of Public Health, Boston, United States of America; 12 Department of Oncology, Mayo Clinic, Rochester, United States of America; Duke Cancer Institute, UNITED STATES

## Abstract

**Purpose:**

Genome-wide-association studies (GWAS) have identified numerous single nucleotide polymorphisms (SNPs) that are associated with an increased risk of breast cancer. Most of these studies were conducted primarily in postmenopausal breast cancer patients. Therefore, we set out to assess whether or not these breast cancer variants are also associated with an elevated risk of breast cancer in young premenopausal patients.

**Methods:**

In 451 women of European ancestry who had prospectively enrolled in a longitudinal cohort study for women diagnosed with breast cancer at or under age 40, we genotyped 44 SNPs that were previously associated with breast cancer risk. A control group was comprised of 1142 postmenopausal healthy women from the Nurses’ Health Study (NHS). We assessed if the frequencies of the adequately genotyped SNPs differed significantly (p≤0.05) between the cohort of young breast cancer patients and postmenopausal controls, and then we corrected for multiple testing.

**Results:**

Genotyping of the controls or cases was inadequate for comparisons between the groups for seven of the 44 SNPs. 9 of the remaining 37 were associated with breast cancer risk in young women with a p-value <0.05: rs10510102, rs1219648, rs13387042, rs1876206, rs2936870, rs2981579, rs3734805, rs3803662 and rs4973768. The directions of these associations were consistent with those in postmenopausal women. However, after correction for multiple testing (Benjamini Hochberg) none of the results remained statistically significant.

**Conclusion:**

After correction for multiple testing, none of the alleles for postmenopausal breast cancer were clearly associated with risk of premenopausal breast cancer in this relatively small study.

## Introduction

Each year, more than 10 000 women are diagnosed with breast cancer before the age of 40 in the United States alone [1, 2], and breast cancer is the most common cause of cancer-related death in women of this age [3]. For unknown reasons, young women are more likely to develop poorly differentiated tumors with low estrogen receptor expression [4–6]. Certain risk factors for premenopausal breast cancer have been shown to differ from those for postmenopausal breast cancer (e.g., thin body habitus increases risk of premenopausal breast cancer but reduces risk of postmenopausal breast cancer), but others (e.g., family history and deleterious *BRCA* mutations) are linked to risk during both periods of life [7–9].

While some cases of both premenopausal and postmenopausal breast cancer are explained by strong environmental exposures (e.g. mediastinal radiation) or mutations in cancer-predisposing genes such as *BRCA* and *p53*, the majority of women with breast cancer lack an obvious environmental trigger or a known genetic syndrome [3]. Therefore, common genetic variants that may contribute to risk are of interest.

Recently, genome-wide association studies (GWAS) have identified numerous single nucleotide polymorphisms (SNPs) that are associated with an increased risk for the development of breast cancer in mostly postmenopausal patients [10–25]. Because most of these GWAS did not include a substantial number of women who were diagnosed with cancer while premenopausal, it has been unclear if the risk alleles discovered in postmenopausal women also predispose to breast cancer at a young age. Tapper and co-workers investigated 1001 women with early onset non-familial invasive breast cancer (≤ 40 years at diagnosis) and genotyped 206 single nucleotide polymorphisms (SNPs) across 30 candidate genes. This study confirmed previous associations between increased breast cancer risk and SNPs in *CASP8*, *TOX3* and *ESR1*.

In order to investigate if known breast cancer genetic risk variants are also associated with premenopausally diagnosed breast cancer, we assessed the frequency of 44 previously identified SNPs known to be associated with risk of postmenopausal breast cancer in a cohort of 451 patients diagnosed with breast cancer at or under age 40. We included all SNPs that had been shown to be predictive of breast cancer risk at the time this study was planned [11, 12, 14, 15, 17, 20–28] We compared the allele frequencies in our cohort of young women with breast cancer to allele frequencies observed in women without evidence of breast cancer.

## Material & methods

### Young patients with breast cancer

The Young Women’s Breast Cancer Study is an ongoing prospective cohort study established to explore biological, medical, and psychosocial aspects of breast cancer in women diagnosed with breast cancer at or under 40 years of age (NCT01468246). Between November 2006 and December 13, 2012, 1463 women were invited to participate from eleven sites in Massachusetts, one site in Denver, Colorado, and one site in Toronto, Canada. Eligibility requirements included age 40 years or younger and diagnosis with stage 0–4 breast cancer less than six months prior to enrollment.

After 915 patients signed informed consent in person or by mail between 11/1/2006 and 12/13/2012, they received mailed surveys that included questions about sociodemographic information and medical history. In addition, medical record and central pathology review were used to obtain data on tumor stage, grade, ER/PR expression, and Her2/neu (human epidermal growth factor receptor 2) overexpression. Blood samples were collected at enrollment, one year after diagnosis, and four years after diagnosis. For the present study, only one blood sample per patient was genotyped.

Participants with non-invasive breast cancer (n = 32), as well as those with missing participant information (n = 31), were excluded from the study. In total, 451 patients with stage 1–4 breast cancer were eligible for inclusion in our analysis. This study was approved by the Institutional Review Board at Dana–Farber/Harvard Cancer Center as well as at other study sites.

### Controls

Comparator genotype data came from 1142 controls who had no history of cancer and were participants in the Nurses’ Health Study (NHS). These were the same controls who were used in the “Cancer Genetic Markers of Susceptibility (CGEMS) Project” [15]. All CGEMS data are available at dbGAP (Study accession number phs000147.v3.p1)[29]. Control blood samples were provided between 1989–1990, and only those not diagnosed with breast cancer during follow-up (until June 1, 2004) were included as controls. In the CGEMS project, 528.173 SNPs were genotyped with Illumina HumanHap550 and then imputed to HapMap 2. All control data were genotyped directly (n = 22) or imputed in our data (n = 15) (Table 1). All controls self-reported as postmenopausal and Caucasian (Southern European/Mediterranean, Scandinavian, other Caucasian); their inferred ancestry through genetic markers was consistent with this self-report.

**Table 1:** Overview of genotyped and imputed data.

SNP	Data
**rs1011970**	genotyped
**rs10263639**	genotyped
**rs10411161**	imputed
**rs10490113**	genotyped
**rs10510102**	genotyped
**rs1092913**	imputed
**rs11249433**	genotyped
**rs11628293**	imputed
**rs12196481**	imputed
**rs13281615**	imputed
**rs13387042**	genotyped
**rs1562430**	genotyped
**rs16886165**	genotyped
**rs1876206**	imputed
**rs1978503**	genotyped
**rs2046210**	imputed
**rs2180341**	imputed
**rs2380205**	genotyped
**rs2936870**	imputed
**rs2981579**	genotyped
**rs2981582**	imputed
**rs3112612**	genotyped
**rs3734805**	genotyped
**rs3757318**	genotyped
**rs3803662**	genotyped
**rs3863436**	imputed
**rs4415084**	genotyped
**rs458685**	imputed
**rs4784227**	imputed
**rs4973768**	genotyped
**rs614367**	genotyped
**rs704010**	genotyped
**rs8170**	genotyped
**rs865686**	genotyped
**rs889312**	imputed
**rs909116**	genotyped

### DNA-Extraction

DNA was extracted from patient whole blood samples using a Qiagen DNA extraction kit (QIAamp DNA Blood Mini kit) according to manufacturer instructions on a Qiacube instrument at the Dana-Farber Cancer Institute Breast Cancer SPORE CORE Laboratory.

### Genotyping

All DNA samples were genotyped using the Sequenom platform. Sequenom probes and primers were purchased via Mass Array Typer 4.0.20 and MySequenom (www.mysequenom.com, Sequenom Inc). Quality control standards were followed for genotyping: 10% of the samples were genotyped twice and showed a concordance rate of 99.9%. Three SNPs were excluded from the analysis due to call rates of <95% and were replaced by proxies [proxy rs11041665 to rs3817198); proxy rs62391594 to rs6556756; proxy rs11628293 to rs999737].

Seven additional SNPs had to be excluded: rs418470 (proxy to rs1926657) and rs62391594 (proxy to rs6556756) because of missing control data; rs930395 (proxy to rs7716600) due to technical problems; rs1154865 because it had strand-ambiguous alleles (C/G); and rs10995194, rs11041665, rs2269336 due to low imputation quality score (r-sq<0.95) in the CGEMS control data. At three loci, SNPs were in Linkage Disequilibrium (LD) with each other (R2>0.9; R2>0.8; R2>0.68) and are highlighted in Table 2. The final data consisted of 37 SNPs tested in 451 cases and 1142 controls. We limited the analysis to European-ancestry patients (n = 451). All SNPs (cases and controls) were in Hardy-Weinberg equilibrium (p-value < = 0.01).

**Table 2:** Associations (described by p-values) between candidate SNPs and breast cancer risk overall and by subtype (before correction for multiple testing).

		HORMONERECEPTOR-STATUS			BRCA		AGE			
	ALL (n = 451)	ER+/PR+, Her2- (n = 193)	Her2+ (n = 134)	TNBC (n = 90)	BRCA+ (n = 38)	BRCA- (n = 223)	<25yrs (n = 10)	26-30yrs (n = 42)	31-35yrs (n = 113)	36-40yrs (n = 286)
**rs1011970**	0.53	0.12	0.78	0.88	0.87	0.49	0.60	0.74	0.63	0.61
**rs10263639**	0.52	0.31	0.72	0.74	0.79	0.96	0.36	1.00	0.39	0.89
**rs10411161**	0.33	0.26	0.45	0.86	0.41	0.46	0.46	0.84	0.96	0.33
**rs10490113**	0.40	1.00	0.16	0.47	0.97	0.41	0.62	0.36	0.96	0.57
**rs10510102**	0.0262	0.28	0.0343	0.21	0.28	0.22	0.61	0.0118	0.10	0.25
**rs1092913**	0.81	0.78	0.92	0.74	0.16	0.54	0.27	0.71	1.00	0.73
**rs11249433**	0.37	0.0193	0.25	1.00	0.63	0.51	0.89	1.00	0.67	0.31
**rs11628293**	1.00	0.77	0.58	0.07	0.71	0.18	0.80	0.40	0.40	0.52
**rs12196481**	0.0214	0.10	0.06	0.25	0.15	0.06	0.75	0.85	0.19	0.0286
**rs13281615**	0.37	0.12	0.81	0.93	0.11	0.38	0.26	0.41	0.60	0.14
**rs13387042**	0.0160	0.0146	0.11	0.93	0.88	0.0082	1.00	0.58	0.0142	0.09
**rs1562430**	1.00	0.17	0.43	0.12	0.80	0.78	0.97	0.78	0.99	0.93
**rs16886165**	0.41	0.16	0.63	0.64	0.56	0.46	0.76	0.40	0.42	0.82
**rs1876206**	0.0160	0.09	0.19	0.10	0.22	0.16	0.12	0.54	0.32	0.0368
**rs1978503**	0.75	0.51	0.80	0.52	0.53	0.89	1.00	0.94	0.41	1.00
**rs2046210**	0.10	0.19	0.54	0.29	0.0068	0.41	0.75	0.52	0.27	0.20
**rs2180341**	0.85	0.99	0.35	0.25	0.66	0.90	0.23	0.75	0.15	0.29
**rs2380205**	0.75	0.51	0.32	0.84	0.71	0.74	1.00	0.96	0.31	0.91
**rs2936870**^**1**^	0.0171	0.0445	0.06	0.32	0.20	0.06	1.00	0.74	0.19	0.0194
**rs2981579**^**1**^	0.0125	0.07	0.0212	0.28	0.26	0.0460	0.82	0.75	0.25	0.0113
**rs2981582**^**1**^	0.08	0.16	0.08	0.77	0.55	0.20	0.97	0.93	0.24	0.07
**rs3112612**	0.37	0.83	0.15	0.59	0.86	0.99	0.40	0.48	0.92	0.48
**rs3734805**^**3**^	0.0122	0.17	0.0294	0.31	0.18	0.17	0.94	0.58	0.0012	0.07
**rs3757318**^**3**^	0.06	0.06	0.23	0.60	0.95	0.53	0.94	1.00	0.0411	0.19
**rs3803662**^**2**^	0.0297	0.13	0.22	0.13	0.44	0.32	0.39	0.16	0.78	0.0329
**rs3863436**	0.24	0.26	0.81	0.18	0.0131	0.72	0.25	0.22	0.43	0.36
**rs4415084**	0.09	0.0163	0.50	0.65	0.52	0.37	0.82	0.99	0.0204	0.32
**rs458685**	0.66	0.70	0.60	0.22	0.49	0.75	0.86	0.79	0.31	0.97
**rs4784227**^**2**^	0.09	0.25	0.23	0.38	1.00	0.48	0.20	0.14	1.00	0.12
**rs4973768**	0.0006	0.0036	0.0016	0.78	0.38	0.0007	1.00	0.38	0.0151	0.0029
**rs614367**	0.25	0.69	0.14	0.78	0.97	0.15	0.66	0.19	0.13	0.91
**rs704010**	0.80	0.35	0.74	0.24	0.80	0.91	0.22	0.26	0.49	0.34
**rs8170**	0.77	0.42	0.40	0.07	0.88	0.34	0.19	0.86	1.00	0.75
**rs865686**	0.10	0.52	0.08	0.31	0.76	0.33	0.97	0.74	0.27	0.09
**rs889312**	0.40	0.20	1.00	0.47	0.53	0.90	0.66	0.30	0.17	0.95
**rs909116**	0.27	0.0213	0.88	0.70	0.79	0.42	1.00	0.06	0.66	0.57
**rs981782**	0.22	0.14	0.14	0.70	0.29	0.23	0.40	0.09	0.0006	0.88

^1), 2)^ and ^3^) in LD with each other

^1)^ R2>0.9

^2)^ R2>0.8

^3)^ R2>0.68

shaded areas: p-values ≤ 0.05 =

### Statistical analysis

For comparison of genotype frequencies, a Chi-Square Test was used. Statistical analyses were performed using R-program (R 3.0.1 GUI, The R Foundation for statistical computing, S.Urbanek & H.-J. Bibiko). All results from the analysis with a p-value <0.05 were considered statistically significant. Correction for multiple testing was performed using the Benjamini-Hochberg method. The threshold for statistical significance based on the multiple tests performed is FDR<0.1. The chi-square test was able to detect a minimal difference in allele frequency of 8.3% with 80% power. The case-control comparison was allele-based. The association is evaluated using multivariate logistic regression, which correspond to the “additive model (odds ratio associated with per allele increase)”. We assessed three tumor biology-derived subgroups in our analysis: 1) patients with ER-positive, PR-positive and Her2-negative breast cancers (n = 193); 2) patients with Her2-positive breast cancers (n = 134); and 3) patients with ER-negative, PR-negative and Her2-negative (triple negative) breast cancers (n = 90). The 34 patients who had other types of tumors (20 with ER-positive, PR-negative, Her2-negative tumors, 6 with ER-negative, PR-positive, Her2-negative, and 8 with missing receptor information) were not included in any subgroup analysis. We also performed subgroup analyses focusing on patients with a known deleterious *BRCA* mutation (*BRCA*+; *BRCA1* n = 27, *BRCA2* n = 11)) and patients known not to carry a deleterious *BRCA* mutation (*BRCA*-) (n = 223). Nine patients were known to have an unclassified variant in one of the *BRCA* genes, 79 patients were not tested, and for 102 the *BRCA*-status data were unknown. Other subpopulations were described by age [≤25yrs (n = 10), 26-30yrs (n = 42), 31-35yrs (n = 113) and 36-40yrs (n = 286)]. We compared each subgroup to the same large control population (n = 1142).

We estimated the number of alternate alleles using the imputed probabilities as follow: P(G = 0) * 0 + P(G = 1) * 1 + P(G = 2) * 2 (0 = AA, 1 = AB, 2 = BB) and rounded the figures, if the results contained fractional parts.

## Results

### Clinical and pathological data

DNA samples from 451 breast cancer patients with a median age at diagnosis of 37 years were genotyped. Please see Table 3 for a summary of the clinical and pathological characteristics of the patient cohort. In the control population of participants from the Nurses Health Study, the median age at the time of DNA collection was 66 years (mean: 65.7 years, range: 44–83 years).

**Table 3:** Description of the young women’s cohort.

	AGE SUBGROUPS (n)									
	17–25 yrs (n = 10)	% of Age group	26–30 yrs (n = 42)	% of Age group	31–35 yrs (n = 113)	% of Age group	36–40 yrs (n = 286)	% of Age group	Total (n = 451)	% of Age group
**CLINICO-PATHOLOGICAL DATA**										
**T**	** **	** **	** **	** **	** **	** **	** **	** **	** **	** **
1	2	20.0	18	42.9	56	49.6	158	55.2	234	51.9
2	4	40.0	18	42.9	40	35.4	96	33.6	158	35.0
3	4	40.0	4	9.5	11	9.7	21	7.3	40	8.9
4	0	0.0	0	0.0	5	4.4	6	2.1	11	2.4
X	0	0.0	1	2.4	1	0.9	4	1.4	6	1.3
missing	0	0.0	1	2.4	0	0.0	1	0.3	2	0.4
**N**										
0	5	50.0	26	61.9	62	54.9	146	51.0	239	53.0
1	4	40.0	9	21.4	33	29.2	104	36.4	150	33.3
2	1	10.0	4	9.5	12	10.6	16	5.6	33	7.3
3	0	0.0	1	2.4	3	2.7	11	3.8	15	3.3
X	0	0.0	1	2.4	3	2.7	8	2.8	12	2.7
missing	0	0.0	1	2.4	0	0.0	1	0.3	2	0.4
**M**										
0	5	50.0	14	33.3	40	35.4	96	33.6	155	34.4
1	1	10.0	2	4.8	6	5.4	17	5.9	26	5.8
X	2	20.0	14	33.3	35	31.0	94	32.9	145	32.2
missing	2	20.0	12	28.6	32	28.3	79	27.6	125	27.7
**Stage**										
1	1	10.0	13	31.0	41	36.3	105	36.7	160	35.5
2	5	50.0	22	52.4	48	42.5	124	43.4	199	44.1
3	3	30.0	5	11.9	18	15.9	38	13.3	64	14.2
4	1	10.0	2	4.8	6	5.3	19	6.6	28	6.2
**Grade**										
1	0	0.0	1	2.4	6	5.3	21	7.3	28	6.2
2	3	30.0	14	33.3	29	25.7	97	33.9	143	31.7
3	7	70.0	27	64.3	77	68.1	166	58.0	277	61.4
X	0	0.0	0	0.0	0	0.0	0	0.0	0	0.0
missing	0	0.0	0	0.0	1	0.9	2	0.7	3	0.7
**Estrogen-receptor**										
positive	7	70.0	28	66.6	81	71.7	190	66.4	306	67.8
negative	3	30.0	14	33.3	32	28.3	95	33.2	144	31.9
missing	0	0.0	0	0.0	0	0.0	1	0.3	1	0.2
**Progesterone-receptor**										
positive	5	50.0	26	61.9	67	59.3	181	63.3	279	61.9
negative	5	50.0	16	38.0	46	40.7	104	36.4	171	37.9
missing	0	0.0	0	0.0	0	0.0	1	0.3	1	0.2
**Her2neu**										
positive	1	10.0	12	28.6	40	35.4	81	28.3	134	29.7
indeterminate	0	0.0	2	4.8	0	0.0	2	0.7	4	0.9
negative	9	90.0	28	66.6	72	63.7	200	69.9	309	68.5
missing	0	0.0	0	0.0	1	0.9	3	1.0	4	0.9
***BRCA*-Testing**										
positive	1	10.0	6	14.3	7	6.2	24	8.4	38	8.4
*BRCA1*	1	10.0	4	9.5	4	3.5	18	6.3	27	6.0
*BRCA2*	0	0.0	2	4.8	3	2.7	6	2.1	11	2.4
unclassified variant	0	0.0	1	2.4	3	2.7	5	1.7	9	2.0
negative	7	70.0	21	50.0	60	53.1	135	47.2	223	49.4
not tested	1	10.0	8	19.0	17	15.0	53	18.5	79	17.5
missing	1	10.0	6	14.3	26	23.0	69	24.1	102	22.6

### SNP frequencies

The evaluation of 37 GWAS-SNPs revealed nine variants that differed significantly between the whole cohort of young breast cancer patients and postmenopausal controls: rs10510102, rs1219648, rs13387042, rs1876206, rs2936870 (proxy for rs2981575), rs2981579, rs3734805, rs3803662 and rs4973768. The directions of these associations were consistent with those in postmenopausal women.

In subgroup analyses, rs13387042 and rs2936870 were only associated with ER-positive, PR-positive, Her2-negative cancers, while rs10510102, rs2981579, rs3734805 were only associated with Her2-positive breast cancers. rs4973768 was associated with both ER-positive, PR-positive, Her2-negative breast cancers and Her2-positive breast cancers. Three SNPs did not appear to be associated with premenopausal breast cancer in any of the subgroups. Results from SNP analyses in the whole cohort as well as in the subgroups are shown in Tables 2 and 4 (Table 2 and Table 4).

**Table 4:** SNP-specific genotype frequencies for cases (YWC) and controls (CGEMS).

	YWC DATA (n = 451)							NHS DATA (= 1142)					
	Allele A	Allele B	AA (n)	AB (n)	BB (n)	missing (n)	AF1	Allele A	Allele B	AA (n)	AB (n)	BB (n)	AF1
**rs1011970**	G	T	300	135	14	2	0.82	G	T	780	328	34	0.83
**rs10263639**	C	T	11	123	315	2	0.16	C	T	24	299	819	0.15
**rs10411161**	G	A	326	107	6	12	0.86	C	T	889	237	16	0.88
**rs10490113**	C	A	2	84	365	0	0.10	C	A	16	227	899	0.11
**rs10510102**	G	A	19	160	270	2	0.22	C	T	37	339	766	0.18
**rs1092913**	G	A	363	82	6	0	0.90	G	A	932	202	8	0.90
**rs11249433**	G	A	76	207	154	14	0.41	G	A	173	544	425	0.39
**rs11628293**	G	A	37	151	263	0	0.25	G	A	76	410	656	0.25
**rs12196481**	C	T	84	206	143	18	0.43	G	A	170	538	434	0.38
**rs13281615**	G	A	68	240	139	4	0.42	G	A	188	533	421	0.40
**rs13387042**	G	A	85	221	143	2	0.44	G	A	275	569	298	0.49
**rs1562430**	C	T	77	233	140	1	0.43	C	T	207	567	368	0.43
**rs16886165**	G	T	8	132	310	1	0.16	G	T	32	284	826	0.15
**rs1876206**	G	A	7	87	355	2	0.11	C	T	27	296	819	0.15
**rs1978503**	G	A	18	124	294	15	0.18	G	A	49	347	746	0.19
**rs2046210**	G	A	173	217	61	0	0.62	G	A	478	542	122	0.66
**rs2180341**	G	A	25	161	262	3	0.24	G	A	70	404	668	0.24
**rs2380205**	C	T	139	222	87	3	0.56	C	T	352	562	228	0.55
rs2936870*	G	A	148	213	88	2	0.57	C	T	435	539	168	0.62
**rs2981579**	C	T	131	213	93	14	0.54	G	A	413	543	186	0.60
**rs2981582**	C	T	155	213	82	1	0.58	G	A	438	538	166	0.62
**rs3112612**	G	A	136	220	92	3	0.55	G	A	386	521	235	0.57
**rs3734805**	C	A	3	86	362	0	0.10	C	A	5	160	977	0.07
**rs3757318**	G	A	366	82	1	2	0.91	G	A	983	154	5	0.93
**rs3803662**	C	T	205	187	44	15	0.68	G	A	597	462	83	0.73
rs3863436*	C	T	167	211	59	14	0.62	C	T	491	503	148	0.65
**rs4415084**	C	T	138	228	83	2	0.56	C	T	429	521	192	0.60
**rs458685**	C	T	18	115	316	2	0.17	G	A	31	301	810	0.16
**rs4784227**	C	T	225	172	38	16	0.71	C	T	634	435	73	0.75
**rs4973768**	G	A	87	224	139	1	0.44	C	T	311	568	263	0.52
**rs614367**	C	T	305	128	6	12	0.84	C	T	837	279	26	0.86
**rs704010**	C	T	158	226	65	2	0.60	C	T	430	537	175	0.61
**rs8170**	G	A	288	134	13	16	0.82	G	A	745	356	41	0.81
**rs865686**	G	T	56	197	196	2	0.34	G	T	173	523	446	0.38
**rs889312**	C	A	30	191	215	15	0.29	C	A	89	445	608	0.27
**rs909116**	C	T	85	229	135	2	0.44	C	T	236	597	309	0.47
**rs981782**	G	T	91	214	143	3	0.44	C	A	266	543	333	0.47

* Proxies (rs2936870*-rs2981575; rs3863436*-rs10871290)

After correction for multiple testing by Benjamini-Hochberg, none of the SNPs were found to be statistically significantly associated with breast cancer risk.

## Discussion

In order to elucidate if the allelic architecture in young women with breast cancer is similar to that in post-menopausal breast cancer, we tested the frequency of previously identified postmenopausal breast cancer risk-associated SNPs in a large cohort of young breast cancer patients. When our initially planned p value threshold of <0.05 was used, nine SNPs (rs10510102, rs1219648, rs13387042, rs1876206, rs2981579, rs3734805, rs3803662, rs4973768, proxy rs2936870) associated with breast cancer in postmenopausal women [11, 12, 16, 20, 22, 23] also appeared to be associated with breast cancer in young women, in the same direction as that observed in postmenopausal women. However, after correction for multiple testing (Benjamini Hochberg) none of the results remained statistically significant.

In the current study, the strongest association with breast cancer in young women was found for rs4973768. Importantly, this SNP was significantly associated with breast cancer in the overall patient cohort as well as in smaller subgroups based on tumor subtype (ER-positive/PR-positive/Her2-negative and Her2-positive breast cancers), *BRCA* status (*BRCA* negative), and age (31-40yrs).

rs4973768 lies in the untranslated region (3’UTR) of the sodium bicarbonate (Na+HCO3-) cotransporter NBCn1 (SLC4A7) [30]. The Nashville Breast Health Study evaluated this SNP in 1511 cases with a mean age of 53.3 years (range 25–75 years) and identified an association with ER-positive breast cancer [31]. Further, a significant association of SNP rs4973768 with increased breast cancer risk was replicated among different ethnicities in mixed age patients [32, 33]. Andersen et al. analyzed whether different SNPs previously identified in GWAS interact with one another and with reproductive and menstrual risk factors in association with breast cancer risk. Including over 1400 European-ancestry women with a median age of 54.5 years, this study confirmed the association of rs4973768 with breast cancer; however, modifications of menstrual and reproductive risk factors associations with breast cancer risk by a polygenic score were not observed [33]. In addition to that, rs4973768 was significantly associated with breast cancer in 477 Chinese, thereby further corroborating the association of rs4973768 in different ethnicities [32]. In contrast to our study, Antoniou and co-workers found an association of rs4973768 with an increased breast cancer risk in *BRCA2* carriers [34], but we likely did not have enough *BRCA2* carriers in our study (n = 11) to find such a link.

rs10510102, rs2981579 and rs3734805 were significantly associated with premenopausal breast cancer in the overall group and in the subgroup with Her2-positive breast cancers. Prior studies have described associations of rs10510102 [12] and rs2981579 (*FGFR2*) [35–37] with breast cancer risk, but not specifically with Her2-positive breast cancers. *FGFR2* (*Fibroblast growth factor receptor 2*) is a member of the tyrosine kinase gene superfamily [38] and is involved cell growth, invasiveness, motility and angiogenesis [38]. Both rs13387042 and rs2936870 (proxy for rs2981575) were significantly associated with risk of premenopausal breast cancer in the overall group and in the Her2-negative subgroup.

rs3803662 on 16q12, located close to *TOX3 (TNRC9*, *CAGF9)* and *LOC643714 [39]*, has been identified as a breast cancer risk allele in various GWAS [11, 16, 22–24] and linked to both ER-positive [22, 40–43] and ER-negative primarily postmenopausal breast cancer [44]. Previous studies suggest that the rs3803662 risk allele may most strongly increase the likelihood of luminal A tumors in postmenopausal breast cancer, and that expression levels of *TOX3* and/or *LOC643714* might influence the progression of breast cancer [39]. Within the current study of younger women, we now report an association of rs3803662 with breast cancer risk in the whole premenopausal patient cohort as well as in the subgroup of patients 36-40yrs, but not in the other subgroups. This is consistent with the prior findings of Tapper et al. [45]

Although the directions of these associations in our cohort were consistent with those observed in postmenopausal women, it has to be emphasized that none of the above mentioned SNPs remained significant after correction for multiple testing, perhaps due to our relatively small sample size. A larger study is needed to more definitively assess the relevance of these SNPs as causal factors in premenopausal breast cancer.

In addition to this limited power, which may have contributed to falsely negative results, our conclusions are limited by the fact that we did not evaluate all of the breast cancer predisposing variants that have now been discovered. For example, Michailidou et al. [19] identified SNPs at 41 new breast cancer susceptibility loci at genome-wide significance in 2013. In cases diagnosed at young age (<40 years), two loci rs2588809 at 14q24.1 (P = 0.001) and rs941764 at 14q32.12 (P = 0.007)) showed higher per-allele ORs. Both SNPs were newly published since we began our work, and therefore not included in our study. The newest breast cancer GWAS was recently published by Michailidou et al. [46].

In addition, earlier this year, Shi et al. [35] used a family-based design to analyze the relationship between breast cancer before age 50 and 77 GWAS-identified risk SNPs. They found 4 SNPs associated with a higher breast cancer risk, two of which, rs3803662 in *TOX3* and rs2981579-A *(FGFR2)*, are consistent with our findings. In our study, one of the SNPs identified to be important by Shi and colleagues, rs999737, had to be excluded due to call rates of <95%. It was replaced by its proxy, rs11628293, which did not appear to be significantly associated with risk of premenopausal breast cancer in our study. We did not assess the fourth SNP found by Shi et al. (rs12662670) because our analysis finished prior to the publication of their work.

A third limitation of this study is that different genotyping platforms were used for controls and patients, potentially introducing bias. In addition, it is possible that allele frequency differences between the control cohort and the premenopausal breast cancer cohort may have been related to age differences between the cohorts (i.e., if a gene predisposes to longevity for reasons other than a reduced risk of breast cancer, we might see a difference in the frequency of a SNP in or around that gene between the cases and controls in our study).

In conclusion, we found that nine SNPs previously associated with postmenopausal breast cancer risk might be associated with breast cancer risk in premenopausal women to some degree, but none of the results remained statistically significant after correction for multiple testing. This adds to the relatively scarce literature evaluating genetic predisposition to young-onset breast cancer [19, 35, 45, 47–50]. Future functional genomics analyses may help us better understand the causes of premenopausal breast cancer, and it will also be important to investigate potential interactions between genetic and environmental risk factors.
