# Correction: Evaluation of significant genome-wide association studies risk—SNPs in young breast cancer patients

**DOI:** 10.1371/journal.pone.0230529

**Published:** 2020-03-12

**Authors:** Michelle Rath, Qiyuan Li, Huili Li, Sara Lindström, Alexander Miron, Penelope Miron, Anne A. Dowton, Meghan E. Meyer, Bryce G. Larson, Mark Pomerantz, Ji-Heui Seo, Laura C. Collins, Hilde Vardeh, Elena Brachtel, Steven E. Come, Virginia Borges, Lidia Schapira, Rulla M. Tamimi, Ann H. Partridge, Matthew Freedman, Kathryn J. Ruddy

The seventh author’s name is spelled incorrectly. The correct name is: Anne A. Dowton.
